# Glass/Polyester Laminates Modified with L-Arginine Phosphate—Effects on the Flammability and Smoke Emission

**DOI:** 10.3390/ma18020286

**Published:** 2025-01-10

**Authors:** Adriana Dowbysz, Mariola Samsonowicz, Bożena Kukfisz, Piotr Koperniak

**Affiliations:** 1 Department of Chemistry, Biology and Biotechnology, Bialystok University of Technology, Wiejska 45A Street, 15-351 Bialystok, Poland; adriana.dowbysz@pb.edu.pl; 2 Institute of Safety Engineering, Fire University, Slowackiego Street 52/54, 01-629 Warsaw, Poland; bkukfisz@apoz.edu.pl; 3 Lukasiewicz Research Network—Institute of Aviation, 110/114 Krakowska Avenue, 02-256 Warsaw, Poland; piotr.koperniak@ilot.lukasiewicz.gov.pl

**Keywords:** glass/polyester laminate, burning behavior, flame retardant, smoke density, L-arginine phosphate

## Abstract

Flammability and smoke generation of glass-fiber-reinforced polyester laminates (GFRPs) modified with L-arginine phosphate (ArgPA) have been investigated. The composition, structure, and thermal degradation processes of ArgPA were assessed by the elemental, FTIR, and thermogravimetric analyses. Flammability and smoke emission of GFRPs varying by different amounts (5–15 wt.%) of bio-based flame retardant (FR) prepared via hand lay-up method were assessed in terms of the limiting oxygen index (LOI) and smoke density tests. It was observed that the addition of ArgPA results in the formation of a charred layer with visible bubbles. The LOI of GFRP with 15 wt.% of ArgPA increased from 20.73 *V*/*V* % (non-modified GFRP) to 24.55 *V*/*V* %, and the material classification was improved from combustible to self-extinguishing. FRs usually increase the specific optical density of smoke, which was also observed for ArgPA-modified GFRPs. However, the specific optical density of smoke at the 4th minute of measurement (D_s(4)_) obtained for ArgPA-modified GFRPs was lower than for GFRPs modified with commercially used APP. TG/FTIR studies of resin modified with ArgPA revealed the presence of phosphorus compounds and non-combustible gases in the decomposition products. Results demonstrate the potential of ArgPA as an effective, bio-based FR for the enhancement of GFRP fire safety.

## 1. Introduction

Today, composites based on unsaturated polyester resin (UPR) are widely used in the shipbuilding, rail, and construction industries due to their high mechanical properties. However, their high flammability and toxic smoke emission during fires pose a serious threat not only to humans, their health, and property but also to the environment [1].

The annual production of flame retardants (FRs) reaches over 2.26 million tons. Although limitations on halogenated FRs have been imposed, they still account for 21% of global production, even though their usage is forbidden in many countries [2].

Therefore, it is advisable to look for other sources of non-toxic and effective FRs, along with their economic availability. For this reason, in recent years, the replacement of halogenated and organophosphorus FRs and others due to the imposed restrictions has led to the development of more sustainable FRs of natural origin. Compounds that exhibit flame-retardant properties usually contain phosphorus, nitrogen, and boron elements [3]. The promising idea is to use nitrogen-rich positively charged amino acids, such as L-arginine (Arg), as they are cheap and available biomass components. Although non-modified L-arginine may not be as effective as commercially available FRs, e.g., aluminum trihydroxide (ATH) or ammonium polyphosphate (APP), their modification with compounds exhibiting flame-retardancy potential in terms of charring or water release abilities may result in obtaining effective bio-based FR.

Arg is a typical green renewable resource [4] mostly obtained via the biotechnological route by microbial fermentation, mainly using *Corynebacterium glutamicum* [5,6]. During Arg combustion, the release of non-combustible gases takes place, suggesting its action in the gas phase in terms of flame-retardant properties. Moreover, Arg also has antibacterial properties [7].

Arg and its derivatives can find application in various materials for flammability reduction. The most recent developments in this field are mainly related to various types of fabrics. Liao et al. [8] grafted the ammonium salt of Arg hexametyhlenephosphonic acid onto cotton fabric, which reached the limiting oxygen index (LOI) value of 45.1 *V*/*V* %, and the peak of heat release rate (pHRR) was reduced from 186.56 kW/m^2^ to 16.05 kW/m^2^. The phosphorylated Arg was grafted onto the lyocell fabric by Chen et al. [4]. The pHRR of modified fabric was significantly reduced by 89.4%. Moreover, the non-combustible gases, such as H_2_O and CO_2_, were released during thermal decomposition, which played an important role in the gas phase. Antibacterial coating consisting of phytic acid and Arg deposited on the polyester–cotton fabric reached an LOI of 32 *V*/*V* %, and a pHRR reduction of 28.97% was observed [9].

However, research on the flame retardancy of Arg and its derivatives as FRs for plastics is limited. He et al. [10] studied the effect of Arg salt of phytic acid (PA) on the flammability of polypropylene. The LOI value at 22 wt.% of additive reached 26 *V*/*V* %, and the pHRR was reduced by 50% (from 1219.3 kW/m^2^ to 609.3 kW/m^2^). The synergistic effect of hydroxylated nano montmorillonite (HNM) and Arg and PA salt on the flammability reduction of polybutylene succinate (PBS) was studied by Kong et al. [11]. The addition of 23.5 wt.% of Arg salt and 1.5 wt.% of HNM increased the LOI to 31.4 *V*/*V* % and reduced the pHRR by 55.8%. Moreover, the modification of PBS improved the impact and bending strength of composites.

The study on the modification of epoxy resins by surface-modified APP containing Arg was conducted by Cheng et al. [12]. With the 25 wt.% additive content, the LOI value reached 34.7%, and the pHRR decreased by 83.5%.

To date, there is little research on the use of Arg and its derivatives, which reduce the flammability of UPRs. The aim of this study is to analyze the influence of L-arginine phosphate (ArgPA) on the flammability of glass-fiber-reinforced polyester laminates (GFRPs). Due to the limited amount of research concerning the smoke emission of GFRPs, smoke density studies have been conducted. Finally, the mechanism of action of ArgPA has been discussed.

## 2. Materials and Methods

### 2.1. Materials

L-arginine (Pol-Aura, Zawroty, Poland) and phosphoric acid (Honeywell, Morristown, NJ, USA) were used for the ArgPA synthesis. APP (Exolit^®^ AP 422) (Clariant AG, Muttenz, Switzerland), as a commercially available FR, was used for the comparative analysis. Orthophthalic, accelerated, and thixotropic UPR was used as a matrix—Polimal^®^ 1094 AWTP-1 (Sarzyna Chemical Sp. z o.o., Nowa Sarzyna, Poland). The UPR was cured with methyl ethyl ketone peroxide Metox-50 (Oxytop, Antoninek, Poland). All chemicals were used without further purification. EM 1002/450/125 chopped strand glass mat (Krosglass S.A., Krosno, Poland) with a basis weight of 450 g/m^2^ was used as reinforcement material.

### 2.2. Methods

#### 2.2.1. ArgPA Synthesis

**Figure 1:**
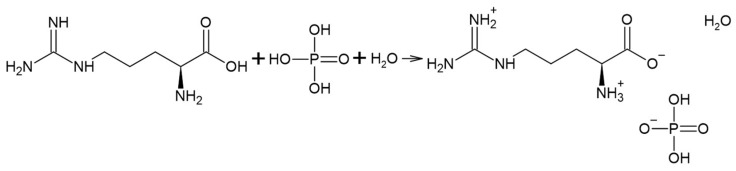
Scheme of ArgPA synthesis.

ArgPA synthesis was conducted as follows according to modified methods [13,14]. L-arginine water solution and phosphoric acid were mixed in a 1:1 molar ratio for 3 h at a temperature of 45 °C. The solution was then evaporated, and obtained ArgPA was dried at a temperature of 80 °C until no further weight loss was observed. The scheme of the synthesis is presented in Figure 1. The reaction yield was 98.66 ± 0.90%.

#### 2.2.2. UPR Casting and Glass/Polyester Laminate Preparation

The UPR was mixed with ArgPA powder to obtain solutions of 5, 10, and 15 wt.% of additive. The solution was mixed with the use of mechanical stirrer (Hei-TORQUE, Heidolph, Schwabach, Germany) for 30 min with a rotational speed of 7000 rpm. Then, the hardener was added (2 wt.% of UPR), and the solution was mixed for another 2 min. The resin was cast into silicone molds and conditioned for 48 h at ambient temperature to obtain castings. The obtained resin castings differed only in terms of the ArgPA content.

UPR solutions for glass/polyester laminate preparation were prepared in the same way as for castings. Laminates with 5, 10, and 15 wt.% of additive were prepared via the hand lay-up method according to previous studies conducted by the authors [15].

#### 2.2.3. Structural, Elemental, and Thermal Analysis

Carbon, hydrogen, and nitrogen content analyses of FRs were carried out using a CHN Analyzer 2400 Series II elemental analyzer (PerkinElmer, Waltham, MA, USA) operating in CHN mode. Samples weighing 2.5 ± 0.5 g, wrapped in tin foil, were pyrolyzed in a stream of oxygen at 905 °C. Measurements were carried out in three repetitions, and standard deviation was calculated.

Particle size distributions of FRs were examined using a Zetasizer Ultra (Malvern Panalytical, Worcestershire, UK). Water solutions (2 mg/mL of FR) were subjected to particle size analysis by volume and intensity.

The FT-IR spectra of the solid FR’s samples were recorded in the range of 400–4000 cm^−1^ with a resolution of 2 cm^−1^ via the ATR multi-reflectance technique with the use of Cary 630 FTIR spectrometer (Agilent Technologies, Santa Clara, CA, USA).

The thermal properties of FRs were investigated using a STA6000 simultaneous thermal analyzer (PerkinElmer, Waltham, MA, USA). A 2.5 ± 0.1 mg sample was placed in a ceramic vessel, and the mass loss and thermal effects were determined under N_2_ atmosphere (flow rate of 50 mL/min) in the temperature range of 25–300 °C (for pure amino acid) or 25–1000 °C, with a heating rate of 10 °C/min.

The thermal properties of UPR were investigated using the same device as for FR analysis. The thermogravimetric (TG) analysis and differential scanning calorimetry (DSC) were conducted in parallel. Analysis of the emitted gaseous products was performed using a Frontier IR spectrometer (PerkinElmer, Waltham, MA, USA) coupled with a STA6000 instrument (PerkinElmer, Waltham, MA, USA) in the range of 450–4000 cm^−1^.

#### 2.2.4. Flammability and Smoke Density of Glass/Polyester Laminates

The minimum concentration of oxygen in the oxygen–nitrogen mixture at which burning process is merely sustained, defined as LOI, was studied in accordance with PN-EN ISO 4589-2:2017-06 Plastics—Determination of ignitability by the oxygen index method—Part 2: Test at room temperature [16] with the use of apparatus for LOI determination (Fire Testing Technology, East Grinstead, UK). A 20 mm × 100 mm laminate sample, vertically positioned in a chimney, was subjected to the flame of a torch for a maximum of 30 s, and the combustion of the material was observed at a gas flow rate of 40 mm/s and a specified volume concentration of oxygen. The measurements were made in three repetitions, and the standard deviation was calculated.

The smoke emission was assessed by the smoke density tests carried out in accordance with PN-EN ISO 5659-2:2017-08 Plastics—Smoke generation Part 2: Determination of optical density by a single-chamber test [17] with the use of a smoke chamber (Sychta Laboratorium, Police, Poland). Laminate sample with dimensions of 75 mm × 75 mm was subjected to an irradiance of 25 kW/m^2^ heat flux with or without a pilot flame. The generated smoke was accumulated in the chamber, and the attenuation of the light beam was measured. The specific optical density of smoke ($D_{s}$) was calculated according to Equation (1) [18]:

(1) $$D_{s}=log\frac{I_{0}}{I}\frac{V}{Al}$$

where V [m^3^] represents chamber volume, A [m^2^] represents the specimen’s exposed area, L [m] represents the path length of the light beam, and $I_{0}$ [−] and I [−] represent the intensities of the light beam before and during the test, respectively. The measurements were made in three repetitions, and the standard deviation was calculated.

#### 2.2.5. Characterization of the Volatile Compounds

The thermal properties of UPR and UPR modified with ArgPA were investigated using the same device as for FRs analysis (STA6000) (PerkinElmer, Waltham, MA, USA). The TG and DSC analyses were conducted in parallel. Analysis of the emitted gaseous products was performed using a Frontier IR spectrometer (PerkinElmer, Waltham, MA, USA) coupled with a STA6000 instrument in the range of 450–4000 cm^−1^.

## 3. Results and Discussion

### 3.1. Elemental, Structural, and Thermal Analysis of FRs

Table 1 presents the calculated and experimental results of the elemental analysis of ArgPA. Results exhibit a high purity of ArgPA obtained from the synthesis. The nitrogen content of ArgPA (18.84 ± 0.22%) is significantly lower compared to Arg (32.10 ± 0.24 [19]). However, phosphorus introduced in its structure (11.3%) may positively affect flame-retardant properties.

FT-IR analysis confirmed the presence of expected functional groups in the ArgPA structure. Detailed data of peaks and corresponding band assignments for Arg and ArgPA are presented in Table 2.

**Figure 2:**
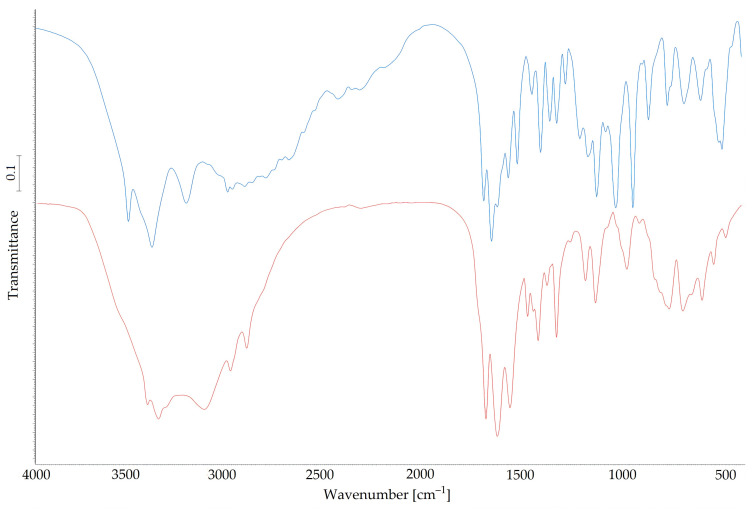
FTIR spectra for Arg (blue) and ArgPA (red).

The hydrated state of water and the ionized form of Arg and phosphate molecules in the ArgPA structure [20] were confirmed by the spectra (Figure 2).

**Table 2:** Wavenumber assignments for Arg and ArgPA.

Arg	Literature	Band Assignment	ArgPA	Literature	Band Assignment
3302, 3355	3302, 3358 [21], 3258–3358 [22]	N–H stretching	3450	3450 [14], 3448 [13], 3451 [20]	O–H stretching
3075	3057 [21]	O–H stretching	3334	3332 [13], 3335 [20]	H_2_O
2865, 2945	2863, 2946 [21]	C–H stretching	3164	3163 [13], 3166 [14], 3168 [20]	NH_3_^+^ asymmetric stretching
1660	1676 [21]	C=O stretching	2959	2959 [14]	C=N–H stretching
1623	1600 [23], 1619 [24]	COO^−^ asymmetric stretching	1690	1691 [13,20]	C–N stretching
1561	1553 [21], 1550 [25], 1556 [24]	N–H bending	1652	1653 [13], 1651 [20]	NH_2_^+^ deformation
1421, 1445, 1473	1374, 1422, 1472 [21], 1465, 1459, 1410 [26]	C–H bending	1569	1573 [13], 1568 [20]	COO^−^ asymmetric stretching
1377	1362 [24]	C–CH in-plane deformation	1524	1527 [13], 1525 [20], 1544 [12]	NH_3_^+^ symmetric deformation
1330	1333 [24]	CH bending	1409	1410 [13], 1408 [20]	COO^−^ symmetric stretching
1187	1186 [24]	NH_2_^+^ rocking	1329	1321 [20], 1335 [13], 1334 [20]	CH_2_ wagging
1137	1137 [21], 1130 [26], 1139 [24]	C–N stretching	1286	1287 [13], 1286 [20]	P–O–H angular
982	979 [21]	O–H bending	1174	1179 [13], 1176 [20]	NH_3_^+^ rocking
772	771 [24]	CH rocking	1132	1136 [13], 1135 [20]	NH_2_ wagging
704	713 [24]	NH_2_ rocking	1037	1040 [13], 1046 [20]	P–O–H group/(PO_4_)
609	623 [24]	CH_2_ rocking	952	952 [13], 950 [20]	P–O–H stretching
551	551, 667 [21], 625 [26]	COO^−^ bending	874	877 [13], 870 [20]	P–O–H stretching
491	493 [24]	COO^−^ rocking	780	790 [13], 782 [20]	NH_2_ rocking
			765	770 [13], 760 [20]	COO^−^ scissoring
			698	699 [13], 696 [20]	NH_2_ out-of-plane bending
			616	615 [13], 614 [20]	OH out-of-plain deformation
			525	525 [20]	PO_4_

Peaks at 3450 cm^−1^ and 3334 cm^−1^ belong to the water of hydration [13], which was suspected in the ArgPA structure. The presence of NH_3_^+^ and NH_2_^+^ groups in the ArgPA structure is confirmed by the peaks at 1524 cm^−1^ (asymmetric stretching) and 1652 cm^−1^ (deformation), respectively [13]. The formation of the phosphate ion in the ArgPA is confirmed by stretching vibrations at 874 cm^−1^, 952 cm^−1^, and 1037 cm^−1^. The intrinsic vibration of this ion can be observed at 525 cm^−1^ [20]. These emerging characteristic peaks found in the ArgPA spectra confirm that phosphate ion was incorporated into the Arg molecule. The obtained results are in good agreement with the previous literature findings.

**Figure 3:**
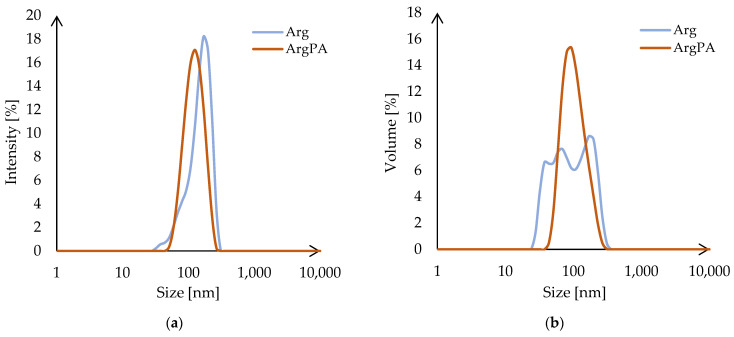
Size distribution by (**a**) intensity and (**b**) volume for the Arg and ArgPA water solutions.

Particle size distributions by intensity and volume are presented in Figure 3a and 3b, respectively. The mean particle size of ArgPA (127.2 nm) is 18% lower compared with the mean particle size of Arg (155.1 nm). According to the size distribution by intensity (Figure 3a), both exhibit homogeneous distribution, with a slightly broader peak observed for Arg (in the range of 32.30—267.20 nm) compared to ArgPA (in the range of 50.79–267.20 nm), indicating a slightly wider size range of particles present in the solution. This is confirmed by the particle size distribution by volume (Figure 3b) due to the presence of several peaks for Arg, corresponding with the total volume of particles in the different size ranges; 12.43 vol. % of Arg particles are lower in size compared to ArgPA.

**Figure 4:**
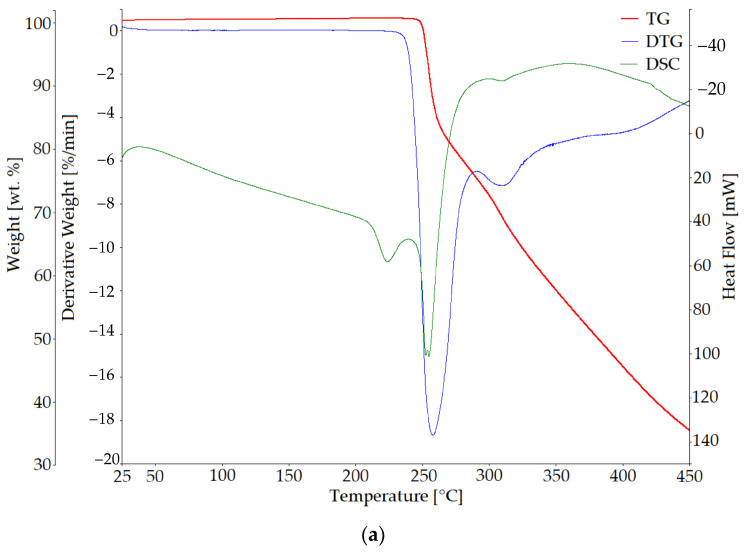

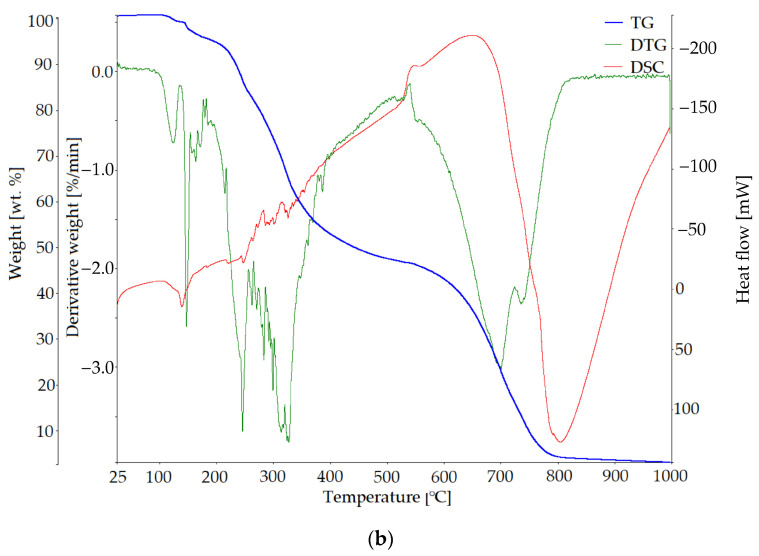
Thermal analysis results of (**a**) Arg and (**b**) ArgPA.

The TG, its first derivative (DTG), and DSC curves of Arg [19] and ArgPA are presented in Figure 4a and 4b, respectively.

Above 100 °C, a water molecule bonds to an ArgPA molecule and is released. The highest rate of mass loss is at 124.21 °C, and the mass loss corresponding to the loss of one water molecule occurs at 127.93 °C. The ArgPA melting point identifies a sharp endothermic peak on the DTG curve at 146.84 °C, which is consistent with the literature findings [27]. A 5% mass loss is recorded at 186.50 °C. The decomposition stage in the temperature range from 182 °C to 540 °C occurs relatively slowly, with a mass loss of 49.11 wt.%. This decomposition stage is associated with the release of carbon dioxide or ammonia [28]. The second stage of decomposition ends at 900 °C, and the residue at 1000 °C is 2.56 wt.%.

The Arg thermal degradation process was described in detail in the previous authors’ work [29]. A comparison of the ArgPA to Arg degradation process can only be provided in the temperature range up to 450 °C due to the way the degradation process of Arg can affect the destruction of equipment [30]. Arg does not contain any water in its structure. The peak at 204 °C (Figure 4a), which does not correspond with any mass loss, is ascribed to the crystal–crystal transition [30]. The second endothermic peak at 226 °C is ascribed to the start of the decomposition process. In the ArgPA DSC curve, two small endothermic peaks are shifted into the higher temperature regime and occur at 282.55 °C and 315.28 °C, respectively. The mass loss at 450 °C is significantly higher for Arg (64.71 wt.%) compared with the mass loss of ArgPA at the same temperature (50.89 wt.%).

### 3.2. Fire Behavior

#### 3.2.1. Limiting Oxygen Index

**Figure 5:**
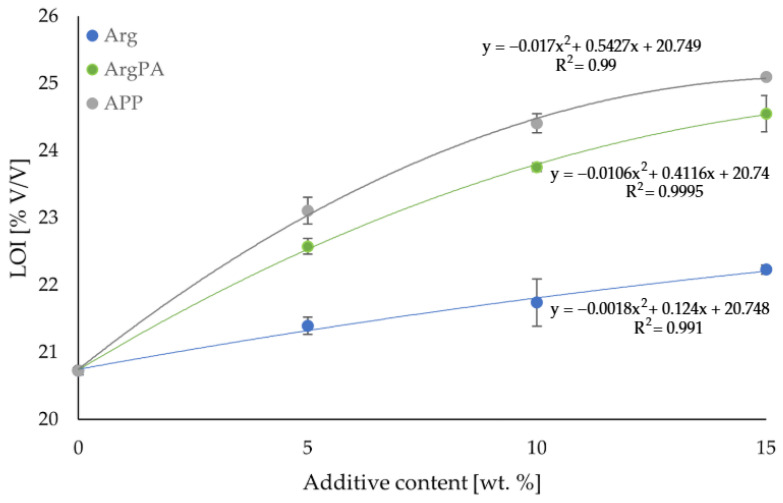
LOI of studied laminates prepared via the hand lay-up method modified with Arg and ArgPA.

The LOI values of Arg-, ArgPA-, and APP-modified laminates are shown in Figure 5. After the resin modification, an increase in LOI values is observed. The relationship between LOI and the amount of additive was non-linear in the studied range, which conformed to the equations shown in Figure 5.

Modification of the laminates with Arg has the lowest influence on the flammability. The highest LOI value is 22.23 ± 0.07 *V*/*V* % at an Arg content of 15 wt.%, which shows a slight increase of 7.24% compared to the non-modified laminate.

The APP-modified laminates at the studied additive content range showed the lowest flammability. At the highest additive content tested (15 wt.%), the LOI values for laminates modified with APP, ArgPA, and Arg additives are 25.10 ± 0.10 *V*/*V* %, 24.55 ± 0.27 *V*/*V* % and 22.23 ± 0.07 *V*/*V* %, respectively. This shows that, compared to unmodified resin laminates, the LOI value increased by 21.08%, 18.23%, and 7.24%, respectively. The LOI values of laminates modified with ArgPA are only slightly lower compared to commercially used APP.

The general formula of APP is (NH_4_)_n+2_P_n_O_3n+1_. The combination of polyhydroxy compound, polyphosphate, and ammonium ions is responsible for the significant flammability reduction [31]. Although ArgPA does not contain ammonium ions in its structure, it does contain amine and phosphate groups, which accounts for some of the structural similarities between these two compounds.

**Figure 6:**
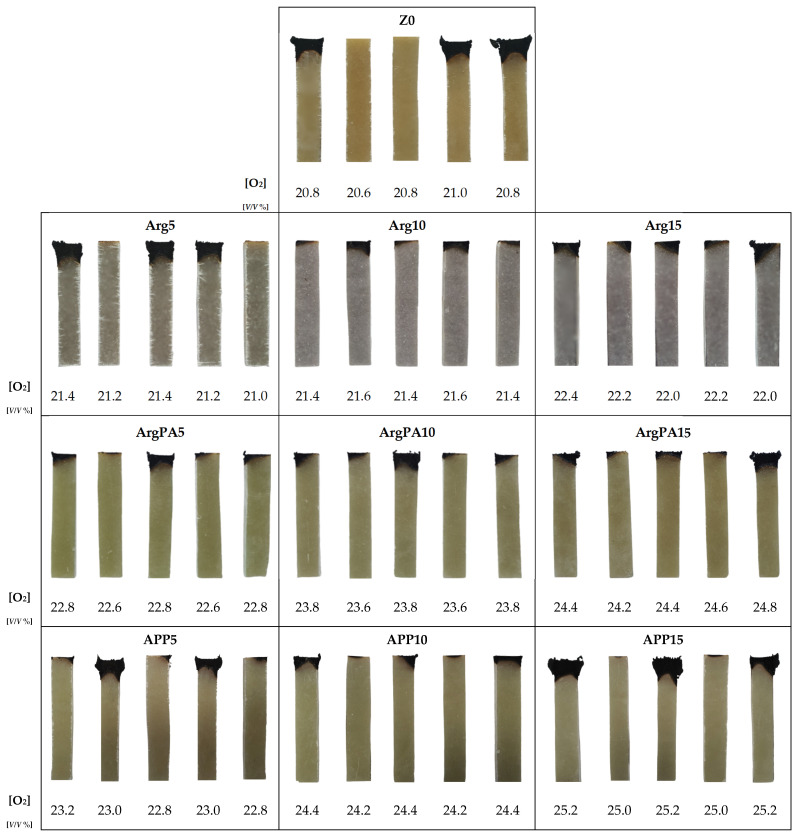
Samples of laminates Z0, Arg, and ArgPA after N_T_ series of LOI tests.

The digital photos of the samples after the NT series of LOI tests are presented in Figure 6.

Based on the previous literature findings, cured UPR exhibits LOI values in the range between 18.9 *V*/*V* % and 21 *V*/*V* % [32,33,34,35,36]. Reinforcing UPR with different types of fibers, e.g., glass, the flammability of UPR is reduced. Interestingly, the addition of FRs to UPR and its further usage in the glass/polyester laminates formation process is not as efficient as the incorporation of FRs to pure UPR. This is due to the difficulties with the development of a stable charring layer caused by the presence of inert fibers [37]. LOI of UPR composites containing several layers of glass fibers may rise up to 24 *V*/*V* % [38].

Although LOI values obtained for ArgPA were not significantly higher compared to pure UPR, the decrease in flammability is clearly visible at a relatively low additive content. For comparison, the addition of 18.5 wt.% of ATH increases the LOI of glass/polyester composite by only two percentage points [39]. An addition of 40 wt.% of ATH has the effect of increasing the LOI to 33 *V*/*V* %. That amount of additive may significantly reduce the mechanical properties of the composites and thus limit their use [40].

#### 3.2.2. Smoke Density

**Figure 7:**
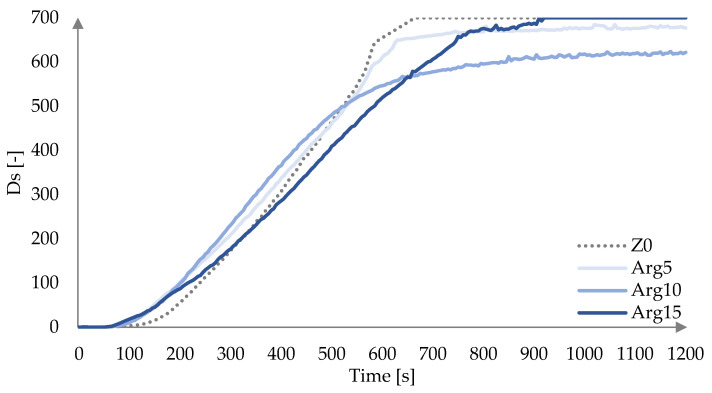
Specific optical density $D_{s}$ as a function of time for laminates modified with Arg at a heat flux of 25 kW/m^2^.

**Figure 8:**
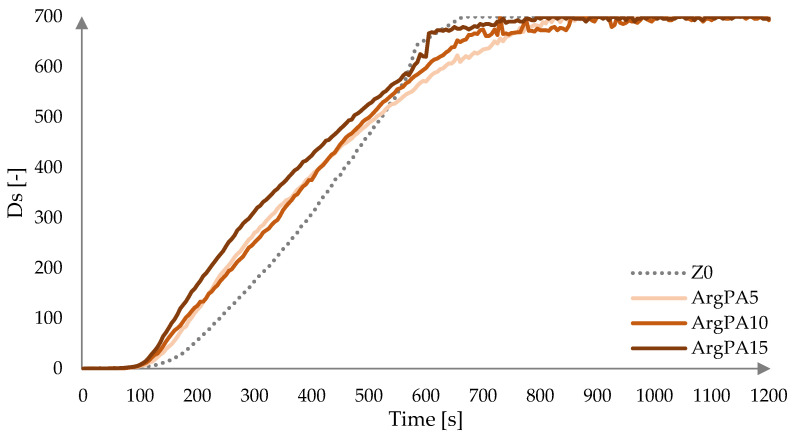
Specific optical density $D_{s}$ as a function of time for laminates modified with ArgPA at a heat flux of 25 kW/m^2^.

**Figure 9:**
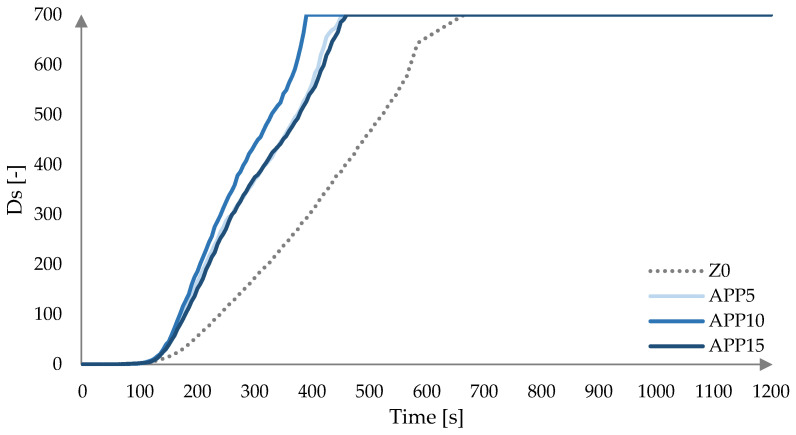
Specific optical density $D_{s}$ as a function of time for laminates modified with APP at a heat flux of 25 kW/m^2^.

The changes of $D_{s}$ in time for laminates modified with FRs are presented in Figure 7, Figure 8 and Figure 9. It was observed that laminates tested under flameless conditions did not ignite. The smoke emission from Arg-modified laminates started earlier than in the case of non-modified laminates (Figure 7). The dynamic increase in $D_{s}$ of the unmodified laminate was recorded after 120 s of measurement, while for the modified laminates, it occurred after 80 s, 90 s, and 75 s, respectively, according to increasing Arg content.

Modification of laminates with Arg causes an increase in the $D_{s}$ from the initial period of the measurement, which is particularly noticeable for laminates modified with 5 wt.% and 10 wt.% additive in the ranges of 80–470 s and 90–525 s, respectively. Modification with 15% wt.% of Arg causes an increase in smoke intensity compared to the unmodified laminate from the initial moment to 300 s of measurement duration. In contrast, in the time range of 350–915 s, the intensity is lower than for the non-modified laminate. The maximum value of the specific smoke optical density ($D_{s\;max}$) is equal to 699.74 for the Arg15 laminate and was reached at 845 ± 106 s of measurement duration. For the unmodified laminate, this value was reached 93 s earlier. The Arg5 and Arg10 laminates show a reduction in smoke intensity compared to the non-modified laminate from 525 s of measurement duration, with the lower smoke intensity of the laminate modified with 10% Arg additive, whose $D_{s\;max}$ value reached 624.76 ± 8.30 s at 1185 ± 21 s.

Modification of the laminates with ArgPA increases the smoke intensity during the initial measurement period, similar to the Arg modification (Figure 8). $D_{s}$ values obtained for laminates containing 5 wt.%, 10 wt.%, and 15 wt.%. of ArgPA are higher compared to the non-modified laminate in the ranges of 100–510 s, 100–550 s, and 95–570 s, respectively. $D_{s\;max}$ of 699.74 was reached by all laminates, with ArgPA modification resulting in a longer time to reach it compared to the Z0 laminate, by 203 s, 143 s, and 93 s, respectively, according to increasing ArgPA content.

Occurring from 700 s to 1200 s of measurement time, the dynamically changing $D_{s}$ values for ArgPA-modified laminates are due to the floating of flocculent particulates produced during decomposition, affecting the interference of the transmittance reading of the light beam transmitted through the chamber.

APP-modified laminates significantly increased the $D_{s}$ values (Figure 9). The decomposition and the smoke emission of the modified laminates were more dynamic, with maximum values reached after 395s for APP10, 465 s for APP5, and 475 (APP15) s.

The initial period of the fire is important from the point of evacuating people. The emitted smoke can significantly reduce the range of visibility, making self-evacuation more difficult. The values for the VOF4 parameter, which is defined as the cumulative value of specific optical density of smoke $D_{s}$, and the $D_{s\;(4)}$ values, which are defined as the specific optical density of smoke at 4 min from the start of the test [41], are presented in Table 3.

Laminates prepared via the contact method differ slightly in resin-to-mat ratio, which may adversely affect the repeatability of the results, as indicated by the standard deviation values obtained (Table 2). It was found that the highest $D_{s\;(4)}$ values were recorded for laminates modified with the commercially used APP. ArgPA had a slightly lower effect on the increase in smoke emission, and the addition of Arg had the least effect on the increase in smoke generation in the first four minutes of measurement compared to unmodified laminate.

The $D_{s}$ values obtained for the studied laminates tested are high, and their maximum values could not be determined due to the capacity of the test chamber. The previous literature reports on epoxy resin containing laminates indicate equally high values for glass-fiber-reinforced composites. Oliva et al. [42] investigated the flammability of composites, where even 18 wt.% of APP and 7 wt.% of dipentaerythritol addition did not reduce $D_{s\;max}$ to values lower than 700.

**Figure 10:**
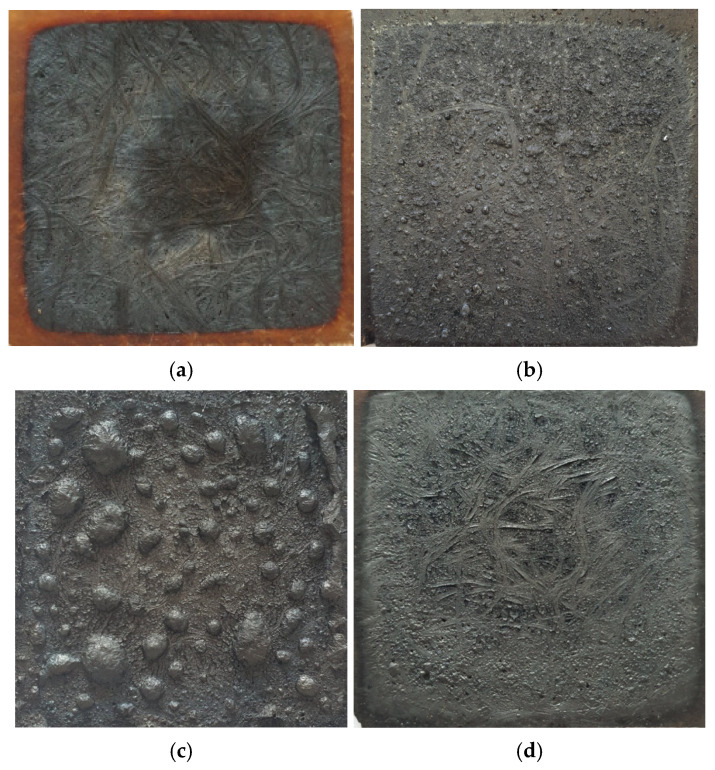
Photographs of the investigated materials after smoke density tests: (**a**) Z0; (**b**) Arg10; (**c**) ArgPA15; (**d**) APP15.

The digital photos illustrating the different burning behaviors of the selected laminates after the smoke density tests have been presented in Figure 10. Fibers of the chopped strand mat become visible in the non-modified laminate and in the APP-modified laminate, indicating a high degree of UPR decomposition. On the surface of Arg- and ArgPA-modified laminates, a charred layer covered with bubbles forms, indicating the release of gases during decomposition. The bubbles’ sizes are much larger for the ArgPA laminate, indicating higher amounts of volatile decomposition products released.

### 3.3. Characterization of Volatile Compounds

**Figure 11:**
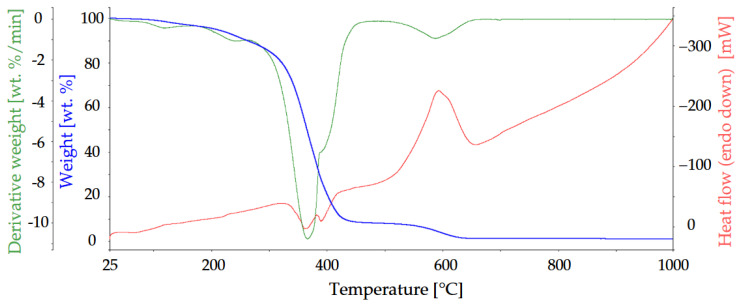
TG, DTG, and DSC curves of UPR.

**Figure 12:**
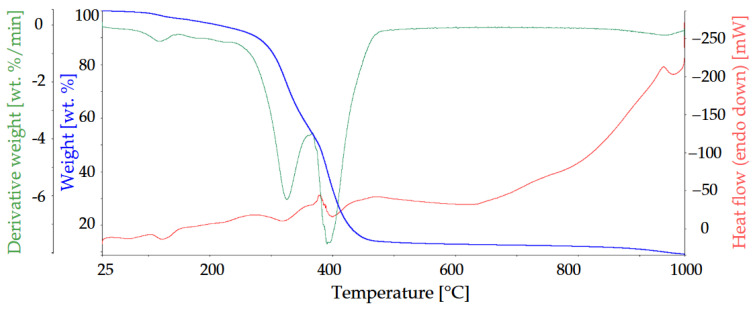
TG, DTG, and DSC curves of UPR modified with 15 wt.% of ArgPA.

Due to the highest flame-retardant effect, ArgPA was the subject of studies on the volatile compounds released during the decomposition process of UPR. The associated thermal effects of UPR and UPR modified with 15 wt.% of Arg are presented in Figure 11 and Figure 12.

The 5 wt.% weight loss of non-modified UPR occurs at 209.16 °C. The main decomposition steps of UPR occur in the temperature range from 100 to 450 °C, with a residue of 8.206 wt.%. The fastest decomposition occurs at 365.99 °C according to the peak in the DSC curve. The last decomposition stage ends at 650 °C. The residue at 1000 °C is 1.12 wt.%.

The 5 wt.% weight loss for the modified resin occurs slightly faster (at 206.72 °C 5 wt.%). The main decomposition occurring between 250 and 450 °C takes place in two steps, with the highest weight loss observed at 323.82 °C (71.764 wt.%) and 396.75 °C (44.592 wt.%), respectively. The residue at 1000 °C is much higher than for the unmodified resin and stands for 8.91 wt.%.

**Figure 13:**
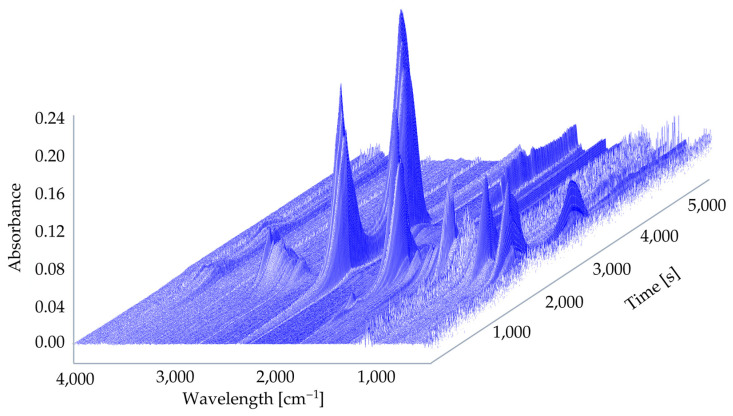
Three-dimensional TG–FTIR spectra of UPR.

**Figure 14:**
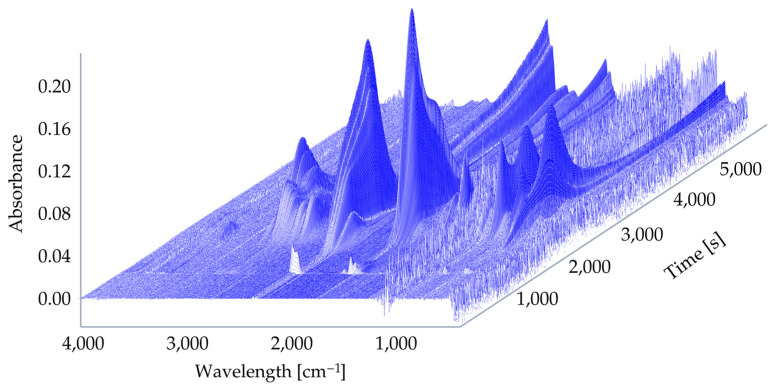
Three-dimensional TG–FTIR spectra of UPR modified with ArgPA.

For composites with a polymeric matrix, gaseous product emission is of great importance. The 3D TG–FTIR spectra of pyrolysis products of UPR and UPR modified with ArgPA during the thermal degradation are shown in Figure 13 and Figure 14.

The FT-IR spectra of volatilized products released at the maximum evolution rate of non-modified laminate at 380 °C, and the peaks assigned for the UPR decomposition were found: 3568–3734 cm^−1^ (H_2_O); 2724–3072 cm^−1^ (C-H, hydrocarbons); 2310 cm^−1^ and 2360 cm^−1^ (CO_2_); 1868 cm^−1^ and 910 cm^−1^ (anhydride); 1736 cm^−1^, 1804 cm^−1^, and 1868 cm^−1^ (C=O, carbonyl compounds); and 1260 cm^−1^ (C-O-C, ester groups). For the FT-IR spectra at 592 °C, the peaks corresponding to the C-H in hydrocarbons, C=O in carbonyl compounds, and C-O-C in ester groups were not detected. However, peaks at 2186 cm^−1^ and 2114 cm^−1^ occurred and were ascribed to the CO release.

Interestingly, based on the spectra of UPR modified with ArgPA obtained at 338 °C, a new peak was detected at 1356 cm^−1^ corresponding with the P=O bond. This suggests the release of compounds containing phosphorus. Their derivatives may terminate free radicals present in the combustion zone during the thermal degradation of UPR.

Based on the spectra of UPR modified with ArgPA obtained at 411 °C, a new peak at 772 cm^−1^ was detected, and the intensity of the peak was at 910 cm^−1^, which is ascribed to the NH_3_ release acting as the blowing agent for the char layer. The increase in the intensity of carbonyl compounds at 1760 cm^−1^ (C=O) was also noted. The comparison of spectra showed no significant difference in the gas phase, suggesting that the addition of ArgPA did not lead to the appearance of many new decomposition products in the gas phase.

All the peaks were ascribed according to the previous literature findings on UPR decomposition [32,43,44,45].

### 3.4. Fire-Retardant Mechanism

**Figure 15:**
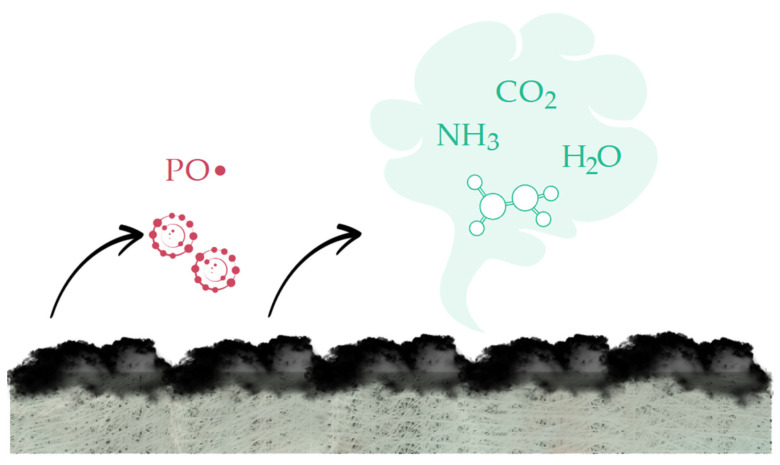
Possible fire-retardant mechanism of ArgPA.

The possible flame-retardant mechanism is presented in Figure 15.

During the thermal degradation, ArgPA releases phosphorus compounds, which further decompose and release phosphorus free radicals, resulting in the occurrence of a quenching effect in the gas phase [11] and in participation of the char layer formation [46]. The amino group of ArgPA undergoes inner cyclization, and further decomposition results in the generation of a great amount of non-combustible gases, such as CO_2_, NH_3_, and H_2_O, which dilute the concentration of flammable gases in the combustion zone [19]. Although the latter two are derived from the addition of ArgPA, the former one (CO_2_) may come from the UPR matrix degradation [1]. However, the intensity of its release is approx. 10 times lower compared to non-modified GFRP.

## 4. Conclusions

In this work, L-arginine phosphate was synthesized, and its structure and thermal stability were characterized. The results of the elemental and FT-IR analyses confirmed the purity and composition of ArgPA. The TG analysis revealed the emission of non-combustible gases such as CO_2_ and NH_3_, as well as lower mass loss as compared to Arg.

Arg, ArgPA, and APP were introduced into the UPR, and glass/polyester laminates were prepared using the hand lay-up method. The flammability test in terms of LOI measurement showed that the highest values were obtained for the 15 wt.% of additives. Laminated modified with Arg proved to be the least flame retardant. The differences in LOI for ArgPA- and APP-modified laminates were not significant, and the LOI was only 2% lower for ArgPA compared to the APP-modified laminate.

Smoke emission studies showed an increase in the $D_{s}$ for ArgPA-modified laminates compared to unmodified laminate. However, the $D_{s\;(4)}$ values were lower (242.65 ± 10.50) than for the APP-modified laminate (252.74 ± 26.16), and the smoke release rate was noticeably lower in the first half of the measurement. The increased smoke rate is related to the release of non-combustible gases such as NH_3_, CO_2_, and H_2_O, as confirmed by TG/FT-IR studies of the modified resins.

The studied GFRPs exhibit lower flammability compared to non-modified laminates and lower smoke density compared to laminates modified with commercially used APP, which may be applied in the shipbuilding and rail industries, especially where a higher level of fire safety is needed. The obtained results presented in this paper exhibit the potential to shape future directions in the search for more sustainable bio-based FRs based on Arg derivatives.

## Figures and Tables

**Table 1:** Results of the elemental analysis of ArgPA.

Compound	Content C [%]		Content H [%]		Content N [%]	
	Calc	Exp	Calc	Exp	Calc	Exp
ArgPA	24.83	24.42 ± 0.20	6.60	6.76 ± 0.09	19.31	18.84 ± 0.22

**Table 3:** VOF4 and $D_{s\;(4)}$ values of studied laminates.

Laminate	VOF4 [−]	$D_{s(4)}$ [−]
Z0	87.58 ± 0.78	94.41 ± 17.85
Arg5	177.08 ± 18.04	145.17 ± 6.86
Arg10	168.88 ± 32.71	153.95 ± 9.53
Arg15	157.75 ± 19.08	117.08 ± 3.04
ArgPA5	185.80 ± 0.59	184.27 ± 8.02
ArgPA10	202.59 ± 65.31	173.91 ± 30.30
ArgPA15	277.62 ± 7.63	242.65 ± 10.50
APP5	240.34 ± 23.56	264.65 ± 29.02
APP10	273.42 ± 1.23	298.61 ± 17.64
APP15	223.23 ± 50.45	252.74 ± 26.16

## Data Availability

The original contributions presented in this study are included in the article. Further inquiries can be directed to the corresponding author.

## Abbreviations

The following abbreviations are used in this manuscript:

GFRP	Glass-fiber-reinforced polyester laminate
ArgPA	L-arginine phosphate
FR	Flame retardant
LOI	Limiting oxygen index
*D*_*s* (4)_	Specific optical density of smoke at 4th minute of measurement
UPR	Unsaturated polyester resin
Arg	L-arginine
ATH	Aluminum trihydroxide
APP	Ammonium polyphosphate
pHRR	Peak of heat release rate
PA	Phytic acid
HNM	Hydroxylated nano montmorillonite
PBS	Polybutylene succinate
TG	Thermogravimetry
DSC	Differential scanning calorimetry
*D_s_*	Specific optical density of smoke
*D_s max_*	Maximum value of the specific smoke optical density

## References

[1] Dowbysz A., Samsonowicz M., Kukfisz B. (2021). Modification of Glass/Polyester Laminates with Flame Retardants. Materials.

[2] Dowbysz A., Samsonowicz M., Kukfisz B. (2022). Recent Advances in Bio-Based Additive Flame Retardants for Thermosetting Resins. Int. J. Environ. Res. Public Health.

[3] Sorathia U., Horrocks A.R., Price D. (2008). 19-Flame Retardant Materials for Maritime and Naval applications. Advances in Fire Retardant Materials.

[4] Chen J., Liu Y., Zhang J., Ren Y., Liu X. (2021). Synthesis of Novel Arginine-Based Flame Retardant and Its Application in Lyocell Fabric. Molecules.

[5] Cheng Y., Zhou Y., Yang L., Zhang C., Xu Q., Xie X., Chen N. (2013). Modification of Histidine Biosynthesis Pathway Genes and the Impact on Production of L-Histidine in Corynebacterium Glutamicum. Biotechnol. Lett..

[6] Park S.H., Kim H.U., Kim T.Y., Park J.S., Kim S.-S., Lee S.Y. (2014). Metabolic Engineering of Corynebacterium Glutamicum for L-Arginine Production. Nat. Commun..

[7] Jin L., Ji C., Chen S., Song Z., Zhou J., Qian K., Guo W. (2023). Multifunctional Textiles with Flame Retardant and Antibacterial Properties: A Review. Molecules.

[8] Liao Y., Chen Y., Zhang F. (2021). A Biological Reactive Flame Retardant for Flame Retardant Modification of Cotton Fabric. Colloids Surf. A Physicochem. Eng. Asp..

[9] Jiang Z., Hu Y., Zhu K., Li Y., Wang C., Zhang S., Wang J. (2022). Self-Assembled Bio-Based Coatings for Flame-Retardant and Antibacterial Polyester–Cotton Fabrics. Text. Res. J..

[10] He S., Gao Y.-Y., Zhao Z.-Y., Huang S.-C., Chen Z.-X., Deng C., Wang Y.-Z. (2021). Fully Bio-Based Phytic Acid–Basic Amino Acid Salt for Flame-Retardant Polypropylene. ACS Appl. Polym. Mater..

[11] Kong Z., Chen Y., Qian L. (2024). The Synergistic Flame Retardant Effect of Biobased Phytic Acid Arginine Salt and Hydroxylated Montmorillonite in Polybutylenes Succinate. Int. J. Biol. Macromol..

[12] Cheng C., Wang Y., Lu Y., Li S., Li H., Yan J., Du S. (2022). Bio-Based Arginine Surface-Modified Ammonium Polyphosphate: An Efficient Intumescent Flame Retardant for Epoxy Resin. RSC Adv..

[13] Sharma S.K., Singh Y., Verma S., Singh M.K., Bartwal K.S., Gupta P.K. (2016). Growth of L-Arginine Phosphate Monohydrate Crystals in Different Orientations to Achieve Isometric Morphology for Device Applications. CrystEngComm.

[14] Premkumar P.S., Shajan X.S., Devadoss H.A. (2010). Optical and Thermal Studies on Oure and Doped L-Arginine Phosphate Crystals. Int. J. Sci. Technol..

[15] Dowbysz A., Samsonowicz M., Markowska D., Jankowski P. (2023). Life Cycle Assessment of Glass/Polyester Laminates Used in the Shipbuilding Industry and Its Fire Behavior. Ekon. I Sr..

[16] (2017). Plastics—Determination of Burning Behaviour by Oxygen Index—Part 2: Ambient-Temperature Test.

[17] (2017). Tworzywa Sztuczne—Wytwarzanie Dymu—Część 2: Oznaczanie Gęstości Optycznej Dymu Metodą Testu Jednokomorowego.

[18] Dowbysz A.M., Samsonowicz M. (2021). Smoke Generation Parameters from the Cone Calorimeter Method and Single-Chamber Test. Environ. Sci. Proc..

[19] Dowbysz A., Samsonowicz M., Kukfisz B. (2024). Thermal Properties of L-Arginine and Its Potential Influence on the Flammability of Unsaturated Polyester Resin. Ideas and Research of Young Scientists.

[20] Haja Hameed A.S., Ravi G., Hossain M.D.M., Ramasamy P. (1999). Growth and Characterisation of L-Arginine Phosphate Family Crystals. J. Cryst. Growth.

[21] Roda A., Santos F., Chua Y.Z., Kumar A., Do H.T., Paiva A., Duarte A.R.C., Held C. (2021). Unravelling the Nature of Citric Acid:L-Arginine:Water Mixtures: The Bifunctional Role of Water. Phys. Chem. Chem. Phys..

[22] Zhang L., Wu C., Gu T., Zhang Y., Liu Y. (2014). Preparation, Characterization and Cytotoxic Activity of Rhein Argininate. Anal. Methods.

[23] Azizi K., Karimi M., Shaterian H.R., Heydari A. (2014). Ultrasound Irradiation for the Green Synthesis of Chromenes Using L-Arginine-Functionalized Magnetic Nanoparticles as a Recyclable Organocatalyst. RSC Adv..

[24] Ramya K., Raja R. (2016). Studies on the Growth and Characterization of L-Arginine Maleate Dihydrate Crystal Grown from Liquid Diffusion Technique. J. Miner. Mater. Charact. Eng..

[25] Sunatkari A.L., Talwatkar S.S., Tamgadge Y.S., Muley G.G. (2015). Synthesis, Characterization and Optical Properties of L-Arginine Stabilized Gold Nanocolloids. Nanosci. Nanotechnol..

[26] Fang H.-Y., Huang W.-M., Chen D.-H. (2019). One-Step Synthesis of Positively Charged Bifunctional Carbon Dot/Silver Composite Nanoparticles for Killing and Fluorescence Imaging of Gram-Negative Bacteria. Nanotechnology.

[27] Mazumder A., Kar T., Siba Prasad Sen Gupta S.P.S.G. (1995). Infrared Spectroscopy and Thermal Studies of As-Grown L-Arginine Phosphate Monohydrate Crystals. Jpn. J. Appl. Phys..

[28] Riscob B., Shakir M., Vijayan N., Maurya K.K., Wahab M.A., Bhagavannarayana G. (2012). Unidirectional Crystal Growth and Crystalline Perfection of L-Arginine Phosphate Monohydrate. J. Appl. Crystallogr..

[29] Bautista Y., Gozalbo A., Mestre S., Sanz V. (2017). Thermal Degradation Mechanism of a Thermostable Polyester Stabilized with an Open-Cage Oligomeric Silsesquioxane. Materials.

[30] Pokorný V., Lieberzeitová E., Štejfa V., Havlín J., Fulem M., Růžička K. (2021). Heat Capacities of L-Arginine, l-Aspartic Acid, l-Glutamic Acid, l-Glutamine, and l-Asparagine. Int. J. Thermophys..

[31] Fink J.K., Fink J.K. (2013). Unsaturated Polyester Resins. Reactive Polymers Fundamentals and Applications.

[32] Salasinska K., Celiński M., Barczewski M., Leszczyński M.K., Borucka M., Kozikowski P. (2020). Fire Behavior of Flame Retarded Unsaturated Polyester Resin with High Nitrogen Content Additives. Polym. Test..

[33] Jiang M., Zhang Y., Yu Y., Zhang Q., Huang B., Chen Z., Chen T., Jiang J. (2019). Flame Retardancy of Unsaturated Polyester Composites with Modified Ammonium Polyphosphate, Montmorillonite, and Zinc Borate. J. Appl. Polym. Sci..

[34] Chen Z., Yu Y., Zhang Q., Chen Z., Chen T., Jiang J. (2019). Preparation of Phosphorylated Chitosan-Coated Carbon Microspheres as Flame Retardant and Its Application in Unsaturated Polyester Resin. Polym. Adv. Technol..

[35] Li J., Gao M., Zheng Y., Guan Y., Yi D. (2020). Effects of Low-Load Boron/Silicon-Based Graphene Oxide on Combustion and Thermal Degradation of Flame-Retardant Unsaturated Polyester Resin. Macromol. Mater. Eng..

[36] Gao W., Yu Y., Chen T., Zhang Q., Chen Z., Chen Z., Jiang J. (2020). Enhanced Flame Retardancy of Unsaturated Polyester Resin Composites Containing Ammonium Polyphosphate and Metal Oxides. J. Appl. Polym. Sci..

[37] Ramadan N., Taha M., La Rosa A.D., Elsabbagh A. (2021). Towards Selection Charts for Epoxy Resin, Unsaturated Polyester Resin and Their Fibre-Fabric Composites with Flame Retardants. Materials.

[38] Zhang S., Chu F., Xu Z., Zhou Y., Hu W., Hu Y. (2021). Interfacial Flame Retardant Unsaturated Polyester Composites with Simultaneously Improved Fire Safety and Mechanical Properties. Chem. Eng. J..

[39] de Freitas Rocha M.A., Landesmann A., da Silva Ribeiro S.P., Martins R.C. (2019). Enhancement of Fire Retardancy Properties of Glass Fibre–Reinforced Polyesters Composites. Fire Mater..

[40] Gunes O.C., Gomek R., Tamar A., Kandemir O.K., Karaorman A., Albayrak A.Z. (2018). Comparative Study on Flame Retardancy, Thermal, and Mechanical Properties of Glass Fiber Reinforced Polyester Composites with Ammonium Polyphosphate, Expandable Graphite, and Aluminum Tri-Hydroxide. Arab. J. Sci. Eng..

[41] Gregory S., Grayson S., Kumar S. (2013). Test Methods and Instrumentation for Assessing Reaction to Fire Properties of Railway Rolling Stock. Probl. Kolejnictwa.

[42] Oliwa R., Oleksy M., Oliwa J., Węgier A., Krauze S., Kowalski M. (2021). Fire Resistant Glass Fabric-Epoxy Composites with Reduced Smoke Emission. Polimery.

[43] Li Y.-M., Hu S.-L., Fang H.-P., Deng Y., Yang C.-D. (2024). Highly-Efficient Flame-Retarding Unsaturated Polyester Resin via the Designation of an Expansive Flame Retardant. Adv. Ind. Eng. Polym. Res..

[44] Dai K., Song L., Jiang S., Yu B., Yang W., Yuen R.K.K., Hu Y. (2013). Unsaturated Polyester Resins Modified with Phosphorus-Containing Groups: Effects on Thermal Properties and Flammability. Polym. Degrad. Stab..

[45] Liu W., Li F., Ge X.G., Zhang Z.J., He J., Gao N. (2016). Effect of DMMP on the Pyrolysis Products of Polyurethane Foam Materials in the Gaseous Phase. IOP Conf. Ser. Mater. Sci. Eng..

[46] Sałasińska K., Celiński M., Mizera K., Barczewski M., Kozikowski P., Leszczyński M.K., Domańska A. (2021). Moisture Resistance, Thermal Stability and Fire Behavior of Unsaturated Polyester Resin Modified with L-Histidinium Dihydrogen Phosphate-Phosphoric Acid. Molecules.

